# A multistep approach to manage Fournier’s gangrene in a patient with unknown type II diabetes: surgery, hyperbaric oxygen, and vacuum-assisted closure therapy: a case report

**DOI:** 10.1186/1752-1947-7-1

**Published:** 2013-01-03

**Authors:** Antonio Luigi Pastore, Giovanni Palleschi, Andrea Ripoli, Luigi Silvestri, Antonino Leto, Domenico Autieri, Cristina Maggioni, Davide Moschese, Vincenzo Petrozza, Antonio Carbone

**Affiliations:** 1 Department of Medico-Surgical Sciences and Biotechnologies, Urology Unit, ICOT, Faculty of Pharmacy and Medicine, Sapienza University of Rome, Via Franco Faggiana 1668, Latina, 04100, Italy; 2 Uroresearch Association, a non-profit association for urology research, Latina, Italy; 3 Department of Medico-Surgical Sciences and Biotechnologies, Faculty of Pharmacy and Medicine, Sapienza University of Rome, Corso della Repubblica 79, Latina, Italy

**Keywords:** Diabetes mellitus, Fournier’s gangrene, Hyperbaric oxygen therapy, Necrotizing fasciitis, Vacuum-assisted closure

## Abstract

**Introduction:**

Fournier’s gangrene is an infectious necrotizing fasciitis of the perineum and genital regions and has a high mortality rate. It is a synergistic infection caused by a mixture of aerobic and anaerobic organisms and predisposing factors, including diabetes mellitus, alcoholism, malnutrition, and low socioeconomic status. We report a case of Fournier’s gangrene in a patient with unknown type II diabetes submitted to 24-hour catheterization 15 days before gangrene onset.

**Case presentation:**

The patient, a 60-year-old Caucasian man, presented with a swollen, edematous, emphysematous scrotum with a crepitant skin and a small circle of necrosis. A lack of resistance along the dartos fascia of the scrotum and Scarpa’s lower abdominal wall fascia combined with the presence of gas and pus during the first surgical debridement also supported the diagnosis of Fournier’s gangrene. On the basis of the microbiological culture, the patient was given multiple antibiotic therapy, combined hypoglycemic treatment, hyperbaric oxygen therapy, and several surgical debridements. After five days the infection was not completely controlled and a vacuum-assisted closure device therapy was started.

**Conclusions:**

This report describes the successful multistep approach of an immediate surgical debridement combined with hyperbaric oxygen and negative pressure wound therapy. The vacuum-assisted closure is a well-known method used to treat complex wounds. In this case study, vacuum-assisted closure treatment was effective and the patient did not require reconstructive surgery. Our report shows that bladder catheterization, a minimally invasive maneuver, may also cause severe infective consequences in high-risk patients, such as patients with diabetes.

## Introduction

We present a case of Fournier’s gangrene in a patient with an unknown type II diabetes mellitus who was given a 24-hour catheterization 15 days before gangrene onset. The aim of this report is to describe our clinical experience regarding patients with Fournier’s gangrene treated with vacuum-assisted closure (VAC) and to clarify the increased risk of a severe infection secondary to minimally invasive urological procedures, such as catheterization, in patients with uncontrolled diabetes.

Fournier’s gangrene, an infective necrotizing fasciitis, is a severe condition with high morbidity and mortality. Predisposing factors include various states of immunosuppression, such as diabetes mellitus, chronic alcoholism, acquired immune deficiency syndrome, and malnutrition [1-3]. The pathology was first described in 1883 by Jean Alfred Fournier who presented five male diabetic patients with fulminating gangrene of the genitalia [4]. Fournier’s gangrene is caused by normal skin commensals of the perineum and genitalia that act synergistically to cause infection and invade the tissue, causing microthrombosis of the small subcutaneous vessels leading to ischemia. Various cytotoxic agents (for example, collagenases, and streptokinases) are released at the gangrene site and cause the progressive destruction of local tissue. Therefore, good management is based on aggressive debridement, broad-spectrum antibiotic therapy, and intensive supportive care.

This report describes the successful use of a multistep approach, including negative-pressure wound therapy, in a case of Fournier’s gangrene developed in a patient with an unknown type II diabetes mellitus.

## Case presentation

In 2011 a 60-year-old Caucasian man was admitted to the Emergency Department of our hospital due to scrotal and perineal pain and fever (39°C). Blood analysis showed the following: leukocytosis (14.47×10^3^/mm^3^), glucose 417mg/dL, creatinine 1.55mg/dL, sodium 136mmol/L, potassium 4.1mmol/L, hematocrit 37.2g/dL, bicarbonate 26mmol/L, total protein 5.1g/dL, and albumin 3.2g/dL. The coagulation parameters were fibrinogen 594mmol/L, partial thromboplastin time 31 seconds, prothombin time-international normalized ratio 1.18, and platelet count 255×10^3^/mm^3^. Ultrasonography of the patient’s scrotum showed the bilateral presence of microbubbles, purulent corpuscular material suggestive for abscess, and a subcutaneous inguinal edema.

At physical examination the patient appeared obese, with a body mass index (BMI) of 31.8; the scrotum presented with a swollen, edematous, emphysematous and crepitant skin with a small circle of necrosis. In his clinical history the patient reported that 15 days prior he had been admitted to the emergency department of another hospital due to abdominal pain and was catheterized for 24 hours, then discharged with the diagnosis of bowel subocclusion.

**Figure 1:**
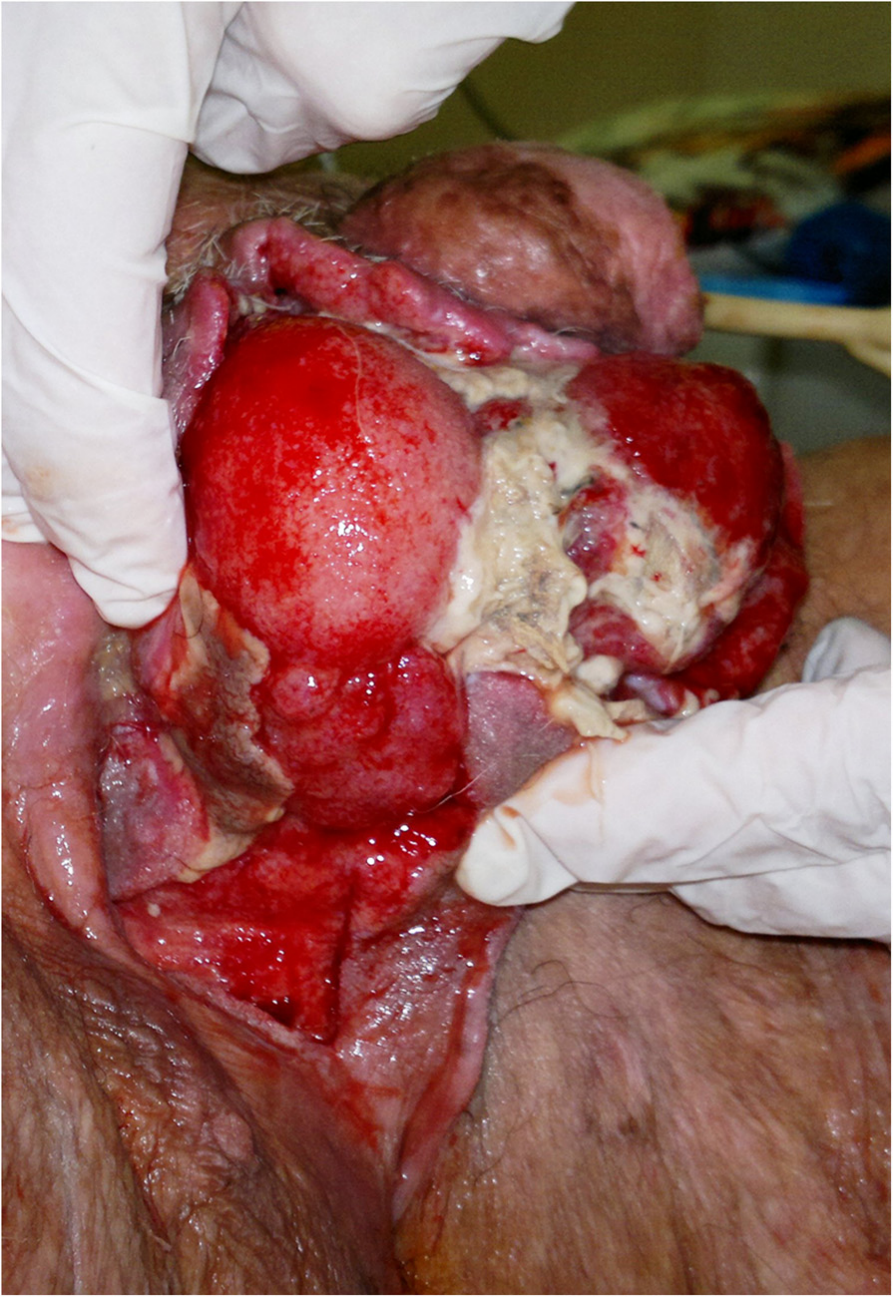
Total exposure of the testes after scrotal debridement.

We submitted the patient to surgical treatment, which consisted of abdominal wall debridement including the inguinal region and scrotum (Figure 1). A lack of resistance along the dartos fascia of the scrotum and Scarpa’s fascia of the lower abdominal wall, as well as minimal bleeding and the presence of gas and pus, were important signs to support the diagnosis of Fournier’s gangrene. Surgical management included wide tissue incisions to allow abundant washing with hydrogen peroxide and saline solution. One subcutaneous trans-scrotal drainage and two subcutaneous suprapubic drainages were inserted. After surgery, an abdominal and pelvic computed tomography (CT) scan showed a right channel inguinal patchy area with predominantly soft tissue density, suggestive for abscess, with evidence of numerous gas microbubbles in contiguity with the scrotum. After the first surgical debridement, a microbiological culture was taken from the tissue sample. A single microorganism was isolated (Group A streptococcus), and antibiotic therapy was adapted to the antibiogram (daptomycin 500mg, piperacillin sodium-tazobactam sodium 4.5mg three times/day, metronidazole 500mg three times/day intravenously) in association with electrolyte replacement.

The diagnosis of diabetes mellitus was confirmed by glycated hemoglobin measurement (7.5%, 59mmol/mol), and five days after surgery, a scheduled combined therapeutic protocol with intermediate acting insulin and oral therapy (metformin 850mg twice/day) finally controlled glucose blood levels.

Debridements were repeated every 24 to 48 hours, and the patient was submitted to 14 sessions of hyperbaric oxygen (HBO) therapy (20 minutes each at 2.4 atmosphere absolute, 100% fraction of inspired oxygen), but the infection was not still completely controlled.

It was then decided to add a new adjuvant treatment – VAC therapy – already in use for diabetic foot ulcers, complex wounds, and abscesses involving the abdominal and chest wall. This VAC therapy (KCI, San Antonio, TX, USA) is a polyurethane sponge cut to the appropriate size and placed over the wound. The sponge, with a suction tube, is covered with a second sterile, adherent, occlusive dressing. Suction is applied to the sponge using a portable pump. The dressing needs to be changed every 24 to 72 hours.

**Figure 2:**
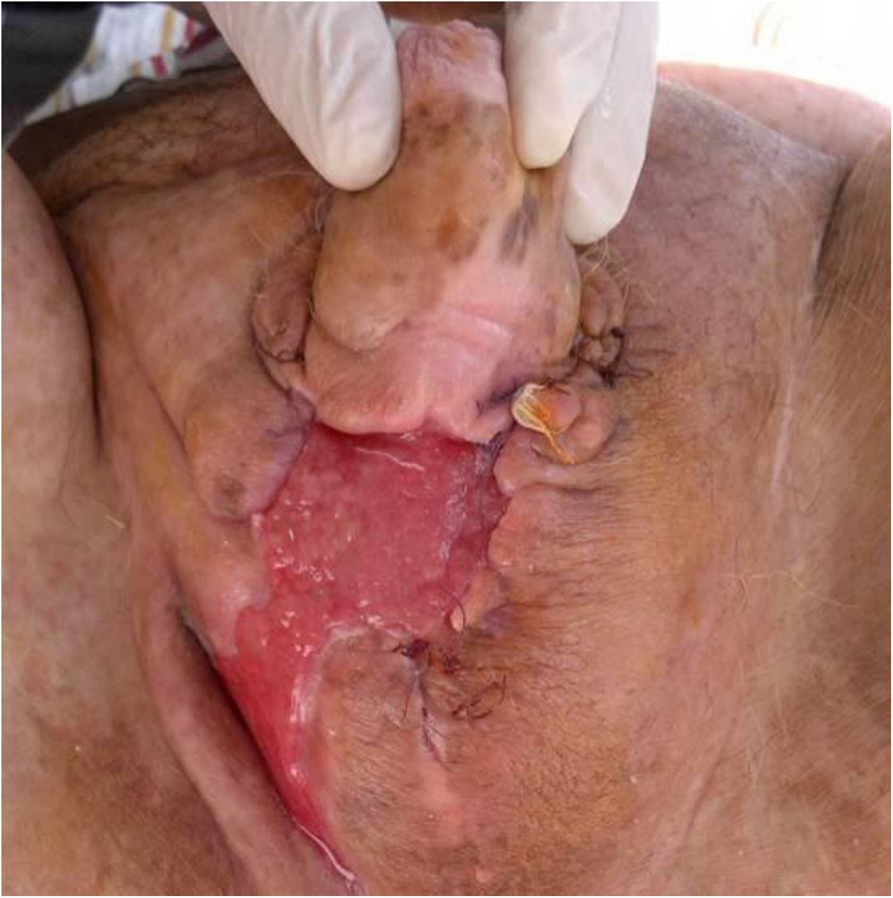
Exposure of the wound at the time of discharge.

The most important benefits of this therapy include a reduction in the wound area together with induction of new granulation tissue, effective wound cleaning, and the continuous removal of wound exudate. After 21 days of VAC therapy, the wound was stabilized, fresh granulation tissue formed with a consequent resorption of the gas microbubbles (confirmed on the next abdominal and pelvic CT), and an important neovascularization of the wound was observed (18 days after hospital admittance). The VAC treatment was so effective that the patient did not require reconstructive surgery. The patient was discharged one month after hospital admission (34 days) with the surgical wound almost completely healed (Figure 2), normal blood glucose levels, and a BMI of 28.7.

## Discussion

Considering the high mortality rate of up to 40%, Fournier’s gangrene, although rare, should be immediately recognized and treated, particularly in patients with predisposing factors, such as autoimmune diseases, alcoholism, low socioeconomic status, intravenous drug use, cancer, and diabetes mellitus [5,6].

Emergency surgical removal of all affected tissue is the primary mandatory treatment even though several repeated debridements might be necessary [5]. However, surgery represents only the first step of a multidisciplinary approach, which involves a urologist, general and plastic surgeon, microbiologist, and nutritionist [7]. Adjuvant treatments include the use of HBO, which has been reported to reduce Fournier’s gangrene mortality compared to exclusive surgical debridement. Optimal tissue oxygenation, obtained by HBO therapy, potentiates the host’s bactericidal mechanisms and wound-healing activity. Furthermore, HBO therapy has a direct toxic effect on anaerobic bacteria [8,9]. Another important adjunctive treatment reported in the literature is VAC, which is currently used for wound complications and is an easy and reliable method that significantly promotes better healing. The negative pressure applied by the device on the wound removes exudates, securely covers the wound, stimulates angiogenesis, and reduces bacterial contamination [10].

Although the use of VAC is not considered a standard approach for Fournier’s gangrene, HBO may have more beneficial effects if combined with a reduction of edema and interstitial pressure achieved by the VAC dressing [11]. Our experience supports this evidence, confirming that the association of VAC therapy with the common therapeutic schedule (surgical debridement, broad-spectrum antibiosis, and HBO) can improve the prognosis of these patients by reducing the recovery time and the need for reconstructive surgery.

## Conclusions

We confirm the benefits of a multistep therapeutic approach (surgery, HBO, and VAC therapy) and suggest this combination as a scheduled effective therapeutic protocol. This clinical case report shows that minimally invasive maneuvers, such as bladder catheterization, may have severe infective consequences in high-risk patients, such as those with diabetes. Surgeons need to be aware of the clinical signs of Fournier’s gangrene and to act promptly given the high mortality rate of the disease.

## Consent

Written informed consent was obtained from our patient for the publication of this case report and any accompanying images. A copy of the written consent is available for review by the Editor-in-Chief of this journal.

## Abbreviations

BMI: Body mass index; CT: Computed tomography; HBO: Hyperbaric oxygen; VAC: Vacuum-assisted closure.

## Competing interests

The authors declare that they have no competing interests.

## Authors’ contributions

All authors equally contributed in writing the manuscript. All authors read and approved the final manuscript. The authors listed below have made substantial contributions to the intellectual content of the paper in the various sections described below: Conception and study design: AR, DA, AL, DM, LS. Acquisition of data: AC, GP, ALP, PV, CM, LS. Analysis and interpretation of data ALP, GP, AR, DA, DM. Drafting of the manuscript: ALP, GP, DM, AR, CM. Critical revision of the manuscript: ALP, GP, AC, PV.
